# REL/DPA/AVI method: a novel approach for rapid detection of carbapenemase-producing Enterobacterales directly from positive blood cultures based on optical density

**DOI:** 10.1128/jcm.01960-24

**Published:** 2025-05-12

**Authors:** Chuwen Zhao, Junqi Zhu, Yanping Xiao, Fuxing Li, Yunwei Zheng, Shumin Gu, Yaping Hang, Qiaoshi Zhong, Longhua Hu

**Affiliations:** 1 Jiangxi Province Key Laboratory of Immunology and Inflammation, Jiangxi Provincial Clinical Research Center for Laboratory Medicine, Department of Clinical Laboratory, The Second Affiliated Hospital, Jiangxi Medical College, Nanchang University 47861, Nanchang, Jiangxi, People’s Republic of China; 2 School of Public Health, Jiangxi Medical College, Nanchang University 47861, Nanchang, Jiangxi, People’s Republic of China; Endeavor Health, Evanston, Illinois, USA

**Keywords:** rapid detection, carbapenemase, *Enterobacterales*, blood cultures, antibiotic resistance, phenotypic testing

## Abstract

The high mortality rate associated with carbapenem-resistant Enterobacterales (CRE), particularly for bloodstream infections (BSI), underscores the urgent need for early identification and differentiation of its resistance mechanisms. In China, traditional phenotypic detection methods for carbapenemases, including the modified Carbapenem Inactivation Method (mCIM), EDTA Carbapenemase Inactivation Method (eCIM), and the carbapenemase inhibitor 3-aminophenylboronic acid (APB) and EDTA enhancement method (APB-EDTA method), are widely used; however, they are time consuming. The relebactam, dipicolinic acid, and avibactam sodium (REL/DPA/AVI) method is a novel phenotypic test for carbapenemase targeting to address these challenges. This method exploits the growth status differences of enzyme-producing bacteria under the combined action of imipenem and enzyme inhibitors (REL, DPA, and AVI) to identify Class A, B, and D carbapenemases at an early stage through optical density (OD) measurements. The REL/DPA/AVI method was optimized and evaluated using 213 contrived (seeded) blood cultures and compared to mCIM/eCIM and APB-EDTA methods. The REL/DPA/AVI method achieved results within 1.5 h (OD measurement) or 2 h (visual observation or OD measurement) from blood culture positivity. Sensitivities of detection of class A, B, D, and A + B carbapenemases at 1.5 h were 97.56% (40/41), 100% (82/82), 71.43% (5/7), and 100% (7/7), respectively. After 2 h, the sensitivity for detecting class D carbapenemases increased to 85.71% (6/7). Conversely, the sensitivities of mCIM/eCIM were 95.83% (46/48) and 97.56% (80/82) for serine β-lactamases and metallo-β-lactamases, respectively. However, the APB-EDTA method demonstrated a sensitivity of 95.1% (39/41), 87.8% (72/82), and 71.43% (5/7) for classes A, B, and A + B carbapenemases, respectively.

**IMPORTANCE:**

The relebactam, dipicolinic acid, and avibactam sodium (REL/DPA/AVI) method has demonstrated significant success in identifying and differentiating carbapenemase-producing Enterobacterales (CPE) from positive blood cultures, exhibiting superior performance compared with existing technologies. Although numerous advanced technologies such as mNGS, Filmarray, Verigene, and NG-Test CARBA 5 DetecTool have been developed for carbapenemase typing of CPE in positive blood cultures, our method is distinguished by a significant economic advantage, with a cost of less than $1 USD per test. This substantial cost-effectiveness underscores the immense potential for widespread clinical applications.

## INTRODUCTION

Since the development of penicillin for treating bacterial infections, frequent and excessive use of antibiotics has led to antimicrobial resistance (AMR), a potential cause of a new public health crisis (1). The most significant global threat is carbapenem-resistant Enterobacterales (CRE), a key priority in the World Health Organization’s updated directory of bacterial priority pathogens (2). Enterobacterales bacteria have a dominant detection frequency of 35.1% in bloodstream infections (BSI), whether community onset or hospital onset, which continues to increase (3). CRE induced by BSI is often associated with significantly high mortality rates, typically ranging from 29% to 46.2%, 2.1 to 3.3 times the mortality rate of carbapenem-susceptible gram-negative bacteria (CS-GNB)-induced BSI (4–7). Numerous studies have indicated that for patients with BSI, delayed appropriate antibiotic treatment after 12 h of blood culture or 48 h of bacteremia can increase the 30 d mortality rate of patients (8, 9).

In recent years, matrix-assisted laser desorption/ionization time-of-flight mass spectrometry (MALDI-TOF MS) has emerged as an effective tool for direct bacterial species identification from positive blood cultures, utilizing either laboratory-developed protocols (e.g., centrifugation-lysis workflows) or commercial kits (10–13). The Rapid MBT Sepsityper kit has demonstrated particularly high performance, achieving up to 97.3% identification accuracy for Enterobacterales (13). However, while rapid species identification is valuable, it alone provides insufficient information for optimizing antimicrobial therapy, especially in cases involving carbapenem resistance, where multiple resistance mechanisms may be involved. The production of carbapenemases represents an important mechanism of CRE (14). Various combinations of antibiotics with carbapenemase inhibitors, such as meropenem-vaborbactam, imipenem-relebactam, ceftazidime-avibactam, and sulbactam-durlobactam, have been developed and used to treat various carbapenem-resistant gram-negative bacteria (CR-GNB). The Infectious Diseases Society of America (IDSA) emphasizes in its treatment guidelines for CRE infections that treatment decisions should be based on the specific type of carbapenemase identified (15). Rapid identification and differentiation of carbapenemases are crucial for timely treatment optimization, patient prognosis improvement, control of CRE spread, and promotion of antimicrobial stewardship.

Currently, carbapenemase-producing Enterobacterales (CPE) detection is primarily divided into phenotypic and genotypic testing. The modified Carbapenem Inactivation Method (mCIM) and ethylenediaminetetraacetic acid (EDTA) Carbapenemase Inactivation Method (eCIM) are phenotypic testing methods endorsed by the Clinical and Laboratory Standards Institute (CLSI) (16). Additionally, the Chinese Expert Consensus guidelines recommend a combination method using 3-aminophenylboronic acid (APB) and EDTA (APB-EDTA method) for carbapenemase detection (17). These methods are characterized by their low cost and high specificity. However, these methods require an overnight incubation step and take a minimum of 48 h to generate results after blood cultures turn positive (17–19). Although Carba NP significantly reduced the time by providing results within 2 h, it exhibited low sensitivity to low-activity OXA-48 (56%) (20). PCR detection has the advantages of high sensitivity, specificity, and speed, and can be directly used for clinical sample testing. However, it is costly, requires special equipment and personnel, and cannot recognize new or mutated genotypes (21).

Recently, Volland developed the BL-DetecTool device to assist in the direct detection of clinical samples using lateral flow immunoassays (LFIAs) such as NG-Test Carba 5, further reducing the test time; however, it can only detect five main carbapenemases (NDM, KPC, IMP, VIM, and OXA-48-like) (22). It cannot recognize some rare (IMI, GES) or mutated genotypes (23). Theoretically, carbapenemase activity-based phenotypic methods can detect almost all categories and variants of carbapenemases (21). Therefore, in this study, we developed and initially validated a targeted carbapenemase phenotypic detection method using contrived (seeded) positive blood culture specimens to classify carbapenemases in Enterobacterales. This method is based on the selective inhibition of different carbapenemase classes by relebactam, dipicolinic acid, and avibactam (REL/DPA/AVI) in the presence of imipenem. When a carbapenemase is effectively inhibited by its specific inhibitor, intact imipenem prevents bacterial growth, resulting in low optical density measurements. In contrast, when carbapenemases remain active, they hydrolyze imipenem, enabling bacterial growth and yielding high optical density readings. Through quantitative analysis of these differential growth patterns using optical density (OD) measurements, this assay can rapidly classify carbapenemase types within 1.5 h after blood culture positivity, offering high specificity and sensitivity while remaining cost-effective.

## MATERIALS AND METHODS

### Bacterial isolates

This study included a total of 213 Enterobacterales isolates (Table 1). Among them, 209 clinical isolates were collected from diverse clinical specimens (sputum, blood, urine, and wound secretions) at the Second Affiliated Hospital of Nanchang University and Shanghai Pulmonary Hospital. Additionally, four reference strains were incorporated: *Klebsiella pneumoniae* ATCC BAA-1705 (KPC carbapenemase producer), *K. pneumoniae* ATCC BAA-2146 (NDM carbapenemase producer), *K. pneumoniae* ATCC BAA-2524 (OXA-48 carbapenemase producer), and *K. pneumoniae* ATCC BAA-1706 (carbapenemase negative). The collection included *Klebsiella pneumoniae*, *Escherichia coli*, *Klebsiella aerogenes*, *Klebsiella oxytoca*, *Enterobacter cloacae complex* (mainly comprising *Enterobacter cloacae*, *Enterobacter asburiae*, *Enterobacter kobei*, and *Enterobacter hormaechei*), *Citrobacter freundii*, *Citrobacter braakii*, *Pantoea agglomerans*, and *Pantoea dispersa*. Preliminary identification of carbapenemase genes in all isolates was performed using PCR and was subsequently validated by DNA sequencing. Based on these results, the collection was divided into the CPE group (*n* = 137) and the non-carbapenemase-producing Enterobacterales group (*n* = 76). The CPE group included the major circulating carbapenemase genotypes in China: class A carbapenemases (*n* = 41, KPC), class B carbapenemases (*n* = 82, NDM, IMP, and NDM + IMP), class D carbapenemases (*n* = 7, OXA-48, OXA-181, and OXA-232), and co-production of two different carbapenemases (*n* = 7, KPC + NDM, and KPC + IMP). The non-carbapenemase-producing Enterobacterales group comprised carbapenem-susceptible Enterobacterales (CSE) and carbapenem-non-susceptible Enterobacterales (CNSE). In this study, CSE were defined as isolates exhibiting full susceptibility to imipenem, meropenem, and ertapenem based on CLSI breakpoints, while CNSE were defined as isolates showing intermediate susceptibility or resistance to any of these carbapenems (24).

**TABLE 1:** Carbapenemase genes and carbapenem MICs of clinical isolates^*a*^

			MIC range (mg/L)		
Carbapenemase type (no.)	Species	β-Lactamase(s) (no.)	IPM	MEM	ETP
Carbapenemase producers (*n* = 137)					
Class A (*n* = 41)	*Klebsiella pneumoniae*	KPC-2 (31)	8–128	16–512	64–512
		KPC-33 (1)	0.125	0.125	0.125
	*Klebsiella aerogenes*	KPC-2 (7)	4–16	8–32	16–128
	*Enterobacter cloacae complex*	KPC-2 (1)	8	16	32
	*Klebsiella pneumoniae*	ATCC BAA-1705 (1)	16	32	64
Class B (*n* = 82)					
NDM	*Klebsiella pneumoniae*	NDM-1 (4)	8–64	0.125–64	16–32
		NDM-3 (1)	16	64	128
		NDM-5 (4)	32–256	0.125–128	4–128
		NDM-16b (1)	16	256	64
		NDM-51 (1)	16	32	64
	*Escherichia coli*	NDM-1 (2)	8–16	8–32	32–64
		NDM-3 (1)	64	32	128
		NDM-4 (1)	8	4	8
		NDM-5 (22)	4–128	16–256	16–512
		NDM-15 (1)	16	256	64
	*Klebsiella oxytoca*	NDM-1 (4)	8–64	8–64	32–128
	*Klebsiella aerogenes*	NDM-1 (1)	32	16	64
		NDM-5 (1)	64	64	128
	*Enterobacter cloacae complex*	NDM-1 (15)	2–32	4–64	4–256
		NDM-5 (5)	4–128	0.125–256	8–512
	*Citrobacter freundii*	NDM-1 (2)	16	16	16–128
	*Citrobacter braakii*	NDM-5 (1)	4	32	64
	*Klebsiella pneumoniae*	ATCC BAA-2146 (1)	64	64	256
IMP	*Klebsiella pneumoniae*	IMP-4 (9)	1–4	1–128	8–512
	*Klebsiella oxytoca*	IMP-4 (2)	2	4–8	8–16
IMP+NDM	*Klebsiella pneumoniae*	NDM-1+IMP-4 (3)	32–64	32	64–128
Class D (*n* = 7)	*Klebsiella pneumoniae*	OXA-48 (1)	16	4	256
		OXA-232 (3)	16–128	32–64	32–512
		OXA-232 (1)	2	1	32
	*Enterobacter cloacae complex*	OXA-181 (1)	1	0.5	4
	*Klebsiella pneumoniae*	ATCC BAA-2524 (1)	2	0.5	2
Double carbapenemases (*n* = 7)	*Klebsiella pneumoniae*	KPC-2+NDM-1 (2)	32–256	64	64–128
		KPC-2+IMP-4 (4)	16–32	16–32	64
	*Klebsiella oxytoca*	KPC-2+IMP-4 (1)	16	16	64
Non-carbapenemase producers (*n* = 76)					
	*Klebsiella pneumoniae*	CMY-170+TEM-179 (1)	0.125	0.125	0.125
		CTX-M-73+OKP-A-11+CMY-170+TEM-207 (1)	0.125	0.125	0.125
		CTX-M-73+SHV-33+CMY-170+TEM-207 (1)	0.25	0.125	0.125
		CTX-M-73+SHV-75+TEM (1)	0.125	0.125	0.125
		CTX-M-73+SHV-81+CMY-170+TEM-207 (1)	0.125	0.125	0.125
		OKP-A-11+CMY-170+TEM-207 (3)	0.125–1	0.125	0.125
		OKP-B-15+TEM-141 (1)	0.125	0.125	0.125
		OKP-B-18+CMY-170+TEM-207 (1)	0.125	0.125	0.125
		OXA-1+SHV-11 (2)	0.5–2	0.125	0.125
		OXA-1+SHV-28+SHV-106+TEM-1+CTX-M-15 (1)	0.125	0.125	0.125
		LEN-16 (1)	0.125	0.125	0.125
		SHV-1+TEM-207 (1)	0.125	0.125	0.25
		SHV-106+CMY-170+TEM-207 (1)	0.125	0.125	0.125
		SHV-106+TEM-207 (1)	0.125	0.125	0.25
		SHV-11+CMY-170+TEM-207 (1)	0.25	0.125	0.25
		SHV-11+TEM-1 (1)	1	0.125	0.5
		SHV-12+TEM-1+CTX-M-14 (1)	0.125	0.125	0.125
		SHV-148+TEM-207 (2)	0.125–0.5	0.125	0.125
		SHV-182+CMY-170+TEM-207 (1)	0.5	0.125	0.5
		SHV-190+TEM-207 (2)	0.125	0.125	0.125
		SHV-28+SHV-106+TEM-1+CTX-M-3 (1)	0.5	1	0.125
		SHV-33+TEM-207 (1)	0.125	0.125	0.125
		SHV-61+CMY-170+TEM-207 (1)	0.125	0.125	0.125
		SHV-71+CMY-170 (1)	0.25	0.125	0.125
		SHV-75+CMY-170+TEM-207 (1)	0.125	0.125	0.125
		SHV-75+TEM-141 (1)	0.125	0.125	0.125
		SHV-81+CMY-170+TEM-207 (1)	0.125	0.125	0.125
		SHV-81+TEM-207 (1)	0.125	0.125	0.125
		SHV-82+CMY-170+TEM-207 (1)	0.125	0.125	0.125
		SHV-89+CMY-170+TEM-207 (2)	0.125	0.125	0.125–0.5
		SHV-11+TEM-209+CTX-M-27+CTX-M-55 (1)	4	2	64
	*Escherichia coli*	CMY-2+TEM-207 (1)	1	0.125	1
		CTX-M-27 (1)	0.125	0.125	0.125
		CTX-M-55 (1)	0.125	2	4
		CTX-M-65+TEM-141 (2)	0.125	0.125	0.125
		CTX-M-73+TEM-207 (1)	0.125	0.125	0.125
		SHV-106+TEM-207 (1)	0.125	0.125	0.125
		SHV-12+CMY-170 (1)	0.125	0.125	0.125
		SHV-148 (1)	0.125	0.125	0.125
		SHV-148+TEM-207 (1)	0.125	0.125	0.125
		TEM-141 (2)	0.125	0.125	0.125
		TEM-168 (1)	0.125	0.125	0.125
		TEM-207 (12)	0.125	0.125	0.125–0.5
		TEM-207+CMY-170 (3)	0.125–1	0.125	0.125
	*Klebsiella oxytoca*	NR (1)	1	0.125	0.5
	*Enterobacter cloacae complex*	TEM-207 (1)	0.25	0.125	0.125
		NR (3)	0.125–1	0.125	0.125–0.5
	*Citrobacter freundii*	CTX-M-73+CMY-170 (1)	0.125	0.125	0.125
		TEM-207+CMY-115 (1)	0.5	0.125	0.125
	*Pantoea agglomerans*	SHV-12 (1)	0.125	0.125	0.125
		NR (1)	0.125	0.125	0.125
	*Pantoea dispersa*	NR (1)	0.125	0.125	0.125
	*Klebsiella pneumoniae*	ATCC BAA-1706 (1)	4	2	8

^*a*^ IPM, imipenem; MEM, meropenem; ETP, ertapenem; NR, no result.

All reference strains were obtained from the American Type Culture Collection (ATCC, USA). All isolates were stored in cryovials at –80°C and were revived and subcultured before testing. Species identification of all isolates was performed using MALDI-TOF MS (VITEK MS IND MALDI TOF, bioMérieux), with *the E. coli* reference strain ATCC 8739 as the quality control strain. The minimum inhibitory concentration (MIC) values for imipenem (Solarbio Science & Technology [Beijing] Co., Ltd.), meropenem (Solarbio Science & Technology [Beijing] Co., Ltd.), and ertapenem (Shanghai Yuanye Bio-Technology Co., Ltd.) were obtained using the broth microdilution method (BMD) according to CLSI guidelines, with the highest test concentration set at 512 mg/L for resistant isolates (MIC ≥ 4 mg/L) and 256 mg/L for non-resistant isolates (MIC < 4), using the *Pseudomonas aeruginosa* ATCC 27853 and *E. coli* ATCC 25922 as the quality control strains (25).

### Preparation of positive blood cultures

Bacterial colonies grown overnight on Columbia agar plates (Thermo Fisher Scientific Biochemical Products [Beijing] Co., Ltd., Beijing, China) were adjusted to a 0.5 McFarland turbidity of 0.9% sodium chloride solution (Jiangxi Kerun Pharmaceutical Co., Ltd.) and diluted 1,000-fold. The diluted bacterial suspension (1 µL) was added to a syringe containing 500 µL of 0.9% sodium chloride solution, resulting in a bacterial concentration of 300 CFU/mL (26), and then inoculated into aerobic blood culture bottles containing sterile human blood (Remel, Santa Fe Trail Drive, Lenexa, KS, USA). After inoculation, the blood culture bottles were incubated in the VersaTREK Automated Microbial Detection System (Thermo Fisher Scientific, Waltham, MA, USA). After the blood culture bottles were positive, 100 µL of blood culture fluid was plated on Columbia agar plates to check the purity of the positive signal.

### Determination of decision time

**Fig 1:**
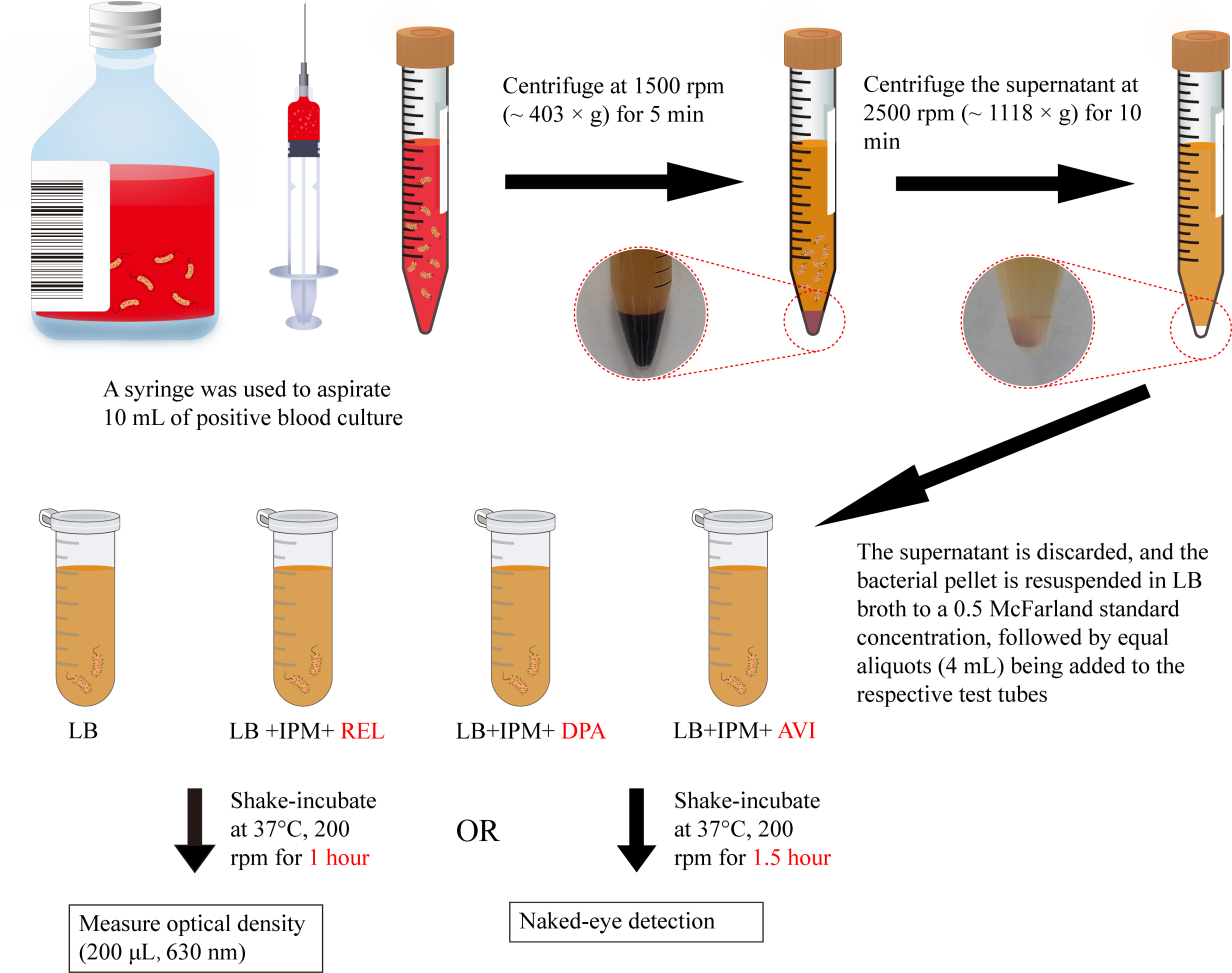
Schematic diagram of the REL/DPA/AVI method for identifying carbapenemase types. LB, LB broth; IPM, imipenem; REL, relebactam; DPA, dipicolinic acid; AVI, avibactam sodium.

A total of 25 randomly selected Enterobacterales isolates, including *K. pneumoniae* (*n* = 5; 1 clinical isolate + 4 reference strains: *K. pneumoniae* ATCC BAA-1705, *K. pneumoniae* ATCC BAA-2146, *K. pneumoniae* ATCC BAA-2524, and *K. pneumoniae* ATCC BAA-1706), *E. coli* (*n* = 5; clinical isolates), *Enterobacter cloacae complex* (*n* = 5; clinical isolates), *Klebsiella aerogenes* (*n* = 5; clinical isolates), and *Klebsiella oxytoca* (*n* = 5; clinical isolates), were processed as described above to prepare positive blood cultures. As shown in Fig. 1, positive blood culture fluid post-positivity signaling (10 mL) was collected in a 15 mL sterile centrifuge tube (Beijing Labgic Technology Co., Ltd, Beijing, China) and was centrifuged at 1,500 rpm (~403 × *g*) for 5 min (to sediment erythrocytes and debris). The supernatant was subsequently centrifuged at 2,500 rpm (~1,118 × *g*) for 10 min (to concentrate the bacteria). The supernatant was discarded, and 0.5 mL (adjust according to the amount of bacterial pellet) of Luria-Bertani broth (LB broth, Solarbio Science & Technology [Beijing] Co., Ltd.) was added to resuspend the white bacterial pellet at the bottom (if there was a red deposition, efforts were made to resuspend only the upper white bacterial pellet). The resuspended pellet was adjusted to a 2 McFarland turbidity bacterial suspension in LB broth for further use. The following procedure was followed to add 1 mL of this resuspended suspension to four 5 mL test tubes as follows: (i) tube 1 contained 3 mL of LB broth; (ii) tube 2 contained 3 mL of LB broth, 3.2 µL of 5 mg/mL imipenem, and 3.2 µL of 10 mg/mL relebactam (Shanghai Macklin Biochemical Co., Ltd.); (iii) tube 3 contained 3 mL of LB broth, 3.2 µL of 5 mg/mL imipenem, and 100 µL of 5 mg/mL DPA (Shanghai Macklin Biochemical Co., Ltd.); (iv) tube 4 contained 3 mL of LB broth, 3.2 µL of 5 mg/mL imipenem, and 6.4 µL of 10 mg/mL avibactam sodium (Solarbio Science & Technology [Beijing] Co., Ltd.). In each test tube, the final bacterial inoculum is approximately 1.5 × 10^8^ CFU/mL (0.5 McFarland standard), with imipenem concentration at 4 mg/L. For the four *K. pneumoniae* reference strains, three additional tubes were established alongside the original four, using the same resuspended reference strain pellet to specifically observe the effects of β-lactamase inhibitors on bacterial growth: (i) tube 5 contained 3 mL of LB broth and 3.2 µL of 10 mg/mL relebactam; (ii) tube 6 contained 3 mL of LB broth and 100 µL of 5 mg/mL DPA; and (iii) tube 7 contained 3 mL of LB broth and 6.4 µL of 10 mg/mL avibactam sodium. The tubes were then placed in a shaking incubator (Changzhou JTLIANGYOU Instrument Co., Ltd.) and incubated at 37°C with shaking at 200 rpm. The optical density changes (OD_630nm_, 200 µL per well) were measured at 0.5 h intervals to determine the earliest judgment time. These were the final optimized test conditions.

### Detection of clinical isolates using REL/DPA/AVI method

Positive blood cultures were prepared for all isolates according to the aforementioned procedures. Typically, 1 mL of the resuspended solution was added to tubes 1–4 and incubated at 37°C with shaking at 200 rpm. The optical density at 1 and 1.5 h is the basis for judgment. Receiver operating characteristic (ROC) curves were used to determine the critical optical density values for discriminating between bacterial growth (where the type of carbapenemase produced by the bacteria was not inhibited by the enzyme inhibitors added to the tubes) and inhibition (where the type of carbapenemase produced by the bacteria was inhibited by the enzyme inhibitors added to the tubes). Based on the obtained cutoff values, growth is denoted with “+” and inhibition with “–.” The following interpretations are considered valid: (i) tubes 1 and 3 are “+,” whereas tubes 2 and 4 are “–,” implying that the isolate may produce class A carbapenemases (KPC, GES, and IMI); (ii) tubes 1, 2, and 4 are “+,” and tube 3 is “–,” suggesting that the isolate may produce class B carbapenemases (NDM, IMP, and VIM); (iii) tubes 1, 2, and 3 are “+,” and tube 4 is “–,” suggesting that the isolate may produce class D carbapenemases (OXA-48, OXA-181, and OXA-232); (iv) tube 1 is “+,” and the remaining tubes are “–,” suggesting that the isolate may be carbapenemase negative; and (v) if all four tubes are “+,” the isolate may be a co-producer of multiple types of carbapenemases or carbapenemase negative (indicating a non-enzyme-mediated mechanism of imipenem resistance), requiring further confirmation. Notably, results cannot be interpreted if tube 1 is judged as “–.”

### Methodological comparison of REL/DPA/AVI method

Carbapenemase phenotypic test methods recommended by the CLSI and the Chinese Expert Consensus, namely, mCIM/eCIM and APB-EDTA methods, were used for enzyme typing of CNSE. Comparative methodological analysis was conducted using the REL/DPA/AVI method. All experimental procedures strictly adhered to the CLSI guidelines and the Chinese Expert Consensus. In accordance with the Chinese Expert Consensus recommendations, the APB-EDTA method uses imipenem susceptibility disks (Oxoid, UK) containing final concentrations of 300 µg/disk for APB and 292 µg/disk for EDTA (17). Quality control was performed using reference strains *K. pneumoniae* ATCC BAA-1705, *K. pneumoniae* ATCC BAA-2146, *K. pneumoniae*, and *K. pneumoniae* ATCC BAA-1706. The criteria for result interpretation are as follows: when the diameter of the zone of inhibition around imipenem disks to which APB and a combination of APB and EDTA were added increased by ≥5 mm compared to the disks without any additions, and the increase was <5 mm in other cases, it was classified as class A carbapenemase; when the diameter of the zone of inhibition around imipenem disks to which EDTA and a combination of APB and EDTA were added increased by ≥5 mm compared to the disks without any additions, and the increase was <5 mm in other cases, it was classified as class B carbapenemase; if the zone diameter of inhibition increased by <5 mm or ≥5 mm in all scenarios, the result was considered uncertain (non-A or non-B class carbapenemase). In this study, if the zone diameter of inhibition around imipenem disks without any additions was ≥23 mm, it was considered carbapenemase negative. For the mCIM procedure following the CLSI guidelines, meropenem susceptibility disks (Oxoid, UK) were immersed in 2 mL of tryptic soy broth (TSB) inoculated with a 1 µL loopful of Enterobacterales. After 4 h of incubation at 35℃, disks were transferred to Mueller-Hinton agar seeded with *E. coli* ATCC 25922 and incubated overnight at 35℃ (27). The eCIM protocol incorporated an additional 20 µL of 0.5 M EDTA (final concentration 5 mM) into the TSB containing a 1 µL loopful of Enterobacterales while maintaining identical procedural steps to mCIM otherwise (27). Results were interpreted in strict compliance with the CLSI guidelines, using *K. pneumoniae* ATCC BAA-1705 and *K. pneumoniae* ATCC BAA-1706 as positive and negative controls, respectively (27).

### Statistical analysis

Data analysis was performed using the Statistical Package for the Social Sciences software (version 20.0). Data were analyzed using normality tests. Data conforming to a normal distribution are expressed as mean ± standard deviation, whereas data not conforming to a normal distribution are represented using interquartile ranges (IQRs). The Mann–Whitney U or Student’s *t*-test was used to compare differences between groups based on whether the data conformed to a normal distribution. ROC curves were used to determine optimal cutoff values. MedCalc (https://www.medcalc.org/) was used to calculate sensitivity, specificity, positive predictive value (PPV), and negative predictive value (NPV). Statistical charts were created using R version 4.4.2 and GraphPad Prism 10.1.2. Statistical significance was set at *P* < 0.05.

## RESULTS

### Principles of REL/DPA/AVI method

The REL/DPA/AVI method rapidly incubated bacteria in identification tubes containing imipenem, specific carbapenemase inhibitors, and LB broth, and monitored changes in optical density to determine the enzyme type (Fig. 1). Differential centrifugation was used to separate most red blood cells and debris from positive blood cultures, concentrating the bacteria and preserving their biological activity. The identification tubes were configured as follows: (i) tube 1 contained only LB broth to assess the viability of the isolated bacteria; (ii) tube 2 contained relebactam and imipenem in the LB broth, where relebactam was used to inhibit class A carbapenemases produced by the bacteria; (iii) tube 3 incorporated DPA and imipenem into the LB broth, with DPA intended to inhibit class B carbapenemases produced by the bacteria; and (iv) tube 4 additionally contained avibactam sodium and imipenem, where avibactam sodium can inhibit both class A and D carbapenemases (OXA-48, OXA-181, and OXA-232) produced by the test strain. After rapid incubation at 37°C and 200 rpm, the optical density was measured at 1 h, and the turbidity was directly observed by the naked eye at 1.5 h to evaluate bacterial growth in each identification tube, thereby determining the type of carbapenemase produced by the test strain.

### Decision time

**Fig 2:**
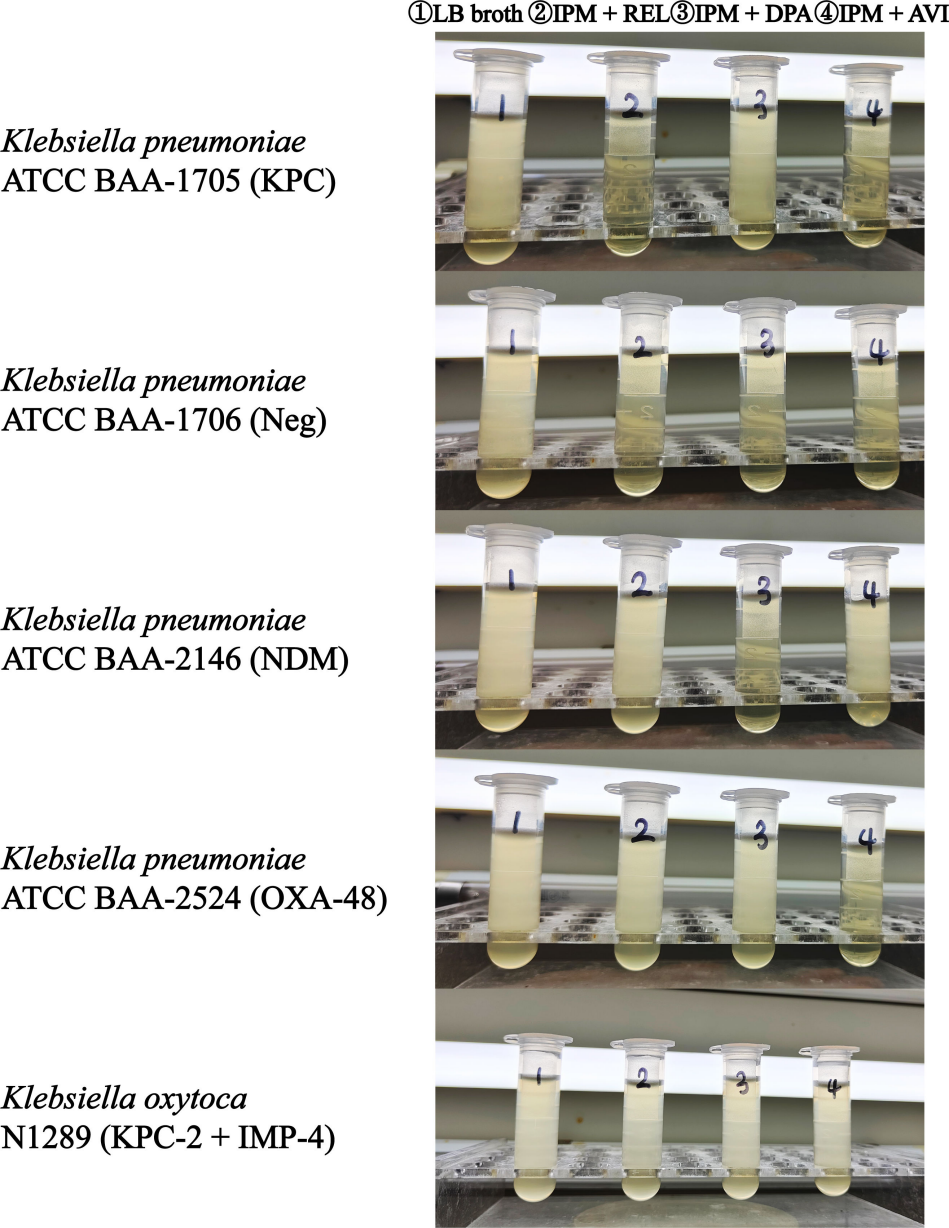
Experimental outcomes after 1.5 h of incubation. IPM, imipenem; REL, relebactam; DPA, dipicolinic acid; AVI, avibactam sodium; Neg, negative.

**Fig 3:**
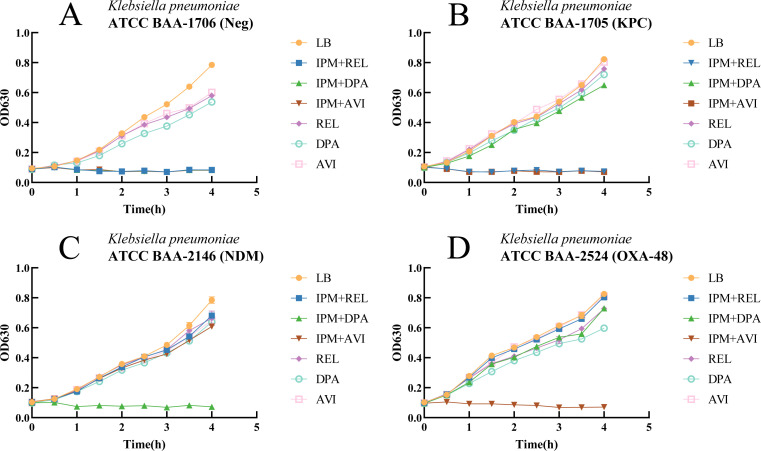
Growth curves of four *Klebsiella pneumoniae* reference strains over 4 h. A–D demonstrate the 4 h optical density changes of carbapenemase-negative, KPC-type, NDM-type, and OXA-48-type carbapenemase-producing *K. pneumoniae* reference strains under different conditions, respectively. OD_630_, optical density at 630 nm; LB, LB broth; IPM, imipenem; REL, relebactam; DPA, dipicolinic acid; AVI, avibactam sodium; Neg, negative.

To investigate the early discrimination of the growth status (growth versus inhibition) of Enterobacterales in the tubes containing different carbapenemase inhibitors, we conducted a time-series analysis of 25 strains of Enterobacterales, including three different types of carbapenemase-positive and one carbapenemase-negative *K. pneumoniae* reference strain. Specifically, the earliest point to distinguish the growth status of Enterobacterales was identified by measuring variations in the optical density of these strains in the four identification tubes over time. The results indicated (Table 2) that enzyme inhibitors significantly suppressed bacterial growth in the inhibition tubes during the 0.5 h of incubation phase, resulting in a significant difference in optical density compared to the growth tubes (*P* < 0.001). However, the optical density difference between the growth and inhibition tubes was negligible due to the short incubation period and the time required for imipenem to exert its effects. As the incubation time was extended to 1 h, the increase in the bacterial reproduction rate and the impact of imipenem led to a further increase in this difference (*P* < 0.001). Notably, an incubation time of 1.5 h was sufficient to distinguish between growth and inhibition in the tubes with the naked eye, manifesting as a contrast between turbidity and clarity (Fig. 2). Consequently, our experimental results suggest that 1 and 1.5 h of incubation are the two key points for the earliest judgment. Furthermore, the 4 h growth curves of the four reference strains in the four identification tubes and three control tubes containing the same inhibitors as the identification tubes but without imipenem were determined to assess the potential impact of enzyme inhibitors on bacterial growth. The experimental results are illustrated in Figure 3. The bacterial growth trend in the control tubes containing only inhibitors was consistent with that in the LB broth tubes without any enzyme inhibitors or antibiotics. Although bacterial growth in LB broth was vigorous, inhibitors exhibited a relatively small effect on bacterial growth (*P* > 0.6), which was insufficient to interfere with the experimental results.

**TABLE 2:** Optical density changes in bacterial growth and inhibition states in identification tubes at various time points^*a*^

Time	OD_630_	*P* value
0 h		
+, mean ± SD	0.0995 (±0.0068)	0.221
–, mean ± SD	0.0977 (±0.0079)	
0.5 h		
+, median (IQR)	0.1360 (0.1265, 0.1460)	<0.001
–, median (IQR)	0.0995 (0.0873, 0.1035)	
1 h		
+, median (IQR)	0.2025 (0.1790, 0.2450)	<0.001
–, mean ± SD	0.0880 (±0.0079)	
1.5 h		
+, median (IQR)	0.2920 (0.2660, 0.3570)	<0.001
–, mean ± SD	0.0835 (±0.0083)	

^*a*^ +, growth; –, inhibition; h, hour; OD_630_, optical density at 630 nm.

### Detection of clinical isolates by REL/DPA/AVI method

The REL/DPA/AVI method was used to identify the 213 Enterobacterales isolates. Figure S1 illustrates the distribution of optical densities for the growth and inhibition states of these isolates after 1 and 1.5 h of incubation. Based on these data, we calculated cutoff values to distinguish between the growth and inhibition states (Table S1). At the 1 h incubation point, an optical density of <0.115 was designated as inhibition (marked as “–”), whereas an optical density of ≥0.115 was designated as growth (marked as “+”). At the 1.5 h incubation point, an optical density of <0.114 was designated as inhibition (marked as “–”), whereas an optical density of ≥0.114 was designated as growth (marked as “+”). Using these cutoff values, we evaluated the performance of the method in detecting class A, B, and D carbapenemases at 1 and 1.5 h of incubation, with the results for each isolate presented in Table S2. Table 3 demonstrates that the sensitivities for detecting class A, B, and D carbapenemases at 1 h were 97.56% (40/41), 100% (82/82), and 71.43% (5/7), respectively. The sensitivity for detecting class D carbapenemases increased to 85.71% (6/7) after 1.5 h of incubation. Regarding the sensitivity and specificity for carbapenemases, the sensitivity and specificity at 1 h were 97.81% (134/137) and 98.68% (75/76), respectively, while the sensitivity increased to 98.54% (135/137) at 1.5 h. Notably, this method also identified seven strains that produced multiple carbapenemases simultaneously (producing KPC-2 and NDM-1 or KPC-2 and IMP-4) and one strain with a non-enzymatic mechanism of imipenem resistance.

**TABLE 3:** Assessment of results from the three phenotypic detection methods^*b*^

Test method	Carbapenemase type	TP	FP	TN	FN	Sensitivity (%)	Specificity (%)	PPV (%)	NPV (%)
APB-EDTA method	Carbapenemase	124	0	4	13	90.51	100.00	100.00	23.53
	A	39	0	104	2	95.12	100.00	100.00	98.11
	B	72	2	61	10	87.80	96.83	97.30	85.92
	A + B	5	0	134	2	71.43	100.00	100.00	98.53
	Others	5	3	131	2	71.43	97.76	62.5	98.50
mCIM/eCIM	Carbapenemase	134	0	4	3	97.81	100.00	100.00	57.14
	Serine-β-lactamase^*a*^	46	1	85	2	95.83	98.84	97.87	97.70
	Metallo-β-lactamase^*a*^	80	0	52	2	97.56	100.00	100.00	96.30
REL/DPA/AVI method for 1 h	Carbapenemase	134	1	75	3	97.81	98.68	99.26	96.15
	A	40	0	172	1	97.56	100.00	100.00	99.42
	B	82	0	131	0	100.00	100.00	100.00	100.00
	D	5	0	206	2	71.43	100.00	100.00	99.04
	Neg	75	1	136	1	98.68	99.27	98.68	99.27
	Double carbapenemases	7	1	205	0	100.00	99.51	87.50	100.00
REL/DPA/AVI method for 1.5 h	Carbapenemases	135	1	75	2	98.54	98.68	99.26	97.40
	A	40	0	172	1	97.56	100.00	100.00	99.42
	B	82	0	131	0	100.00	100.00	100.00	100.00
	D	6	0	206	1	85.71	100.00	100.00	99.52
	Neg	75	1	136	1	98.68	99.27	98.68	99.27
	Double carbapenemases	7	1	205	0	100.00	99.51	87.50	100.00

^*a*^ Calculation does not include double carbapenemases.

^*b*^ A, class A carbapenemases; B, class B carbapenemases; D, class D carbapenemases; A + B, co-producers of class A and B carbapenemases; Neg, carbapenemase negative; TP, true positive; FP, false positive; TN, true negative; FN, false negative; PPV, positive predictive value; NPV, negative predictive value.

### Methodological comparison of REL/DPA/AVI method

In this study, we used phenotypic test methods recommended by the CLSI and the Chinese Expert Consensus to detect carbapenemases in CNSE isolates (Tables 3 and 4; detailed results are provided in Table S3). The sensitivity of the mCIM/eCIM method was 95.83% (46/48) for serine β-lactamases and 97.56% (80/82) for metallo-β-lactamases. Among these, two *K. pneumoniae* isolates producing KPC-33 and OXA-181 and one *C. freundii* isolate producing NDM-1 were misidentified as carbapenemase negative, whereas one *K. oxytoca* isolate producing NDM-1 was misidentified as producing serine β-lactamases. The APB-EDTA method exhibited sensitivities of 95.1% (39/41) for class A carbapenemases, 87.8% (72/82) for class B carbapenemases, 71.43% (5/7) for isolates producing both class A and B carbapenemases, and 71.43% (5/7) for other mechanisms and class D carbapenemases. Notably, one *K. pneumoniae* isolate producing KPC-2 was misidentified as uncertain (all inhibition zone difference of ≥5 mm), two *K. pneumoniae* isolates producing OXA-181 and OXA-232 were misidentified as producing class B carbapenemases, two *K. pneumoniae* and *K. oxytoca* isolates producing KPC-2+IMP-4 were misidentified as uncertain (all inhibition zone difference of <5 mm), and 11 isolates of *K. pneumoniae* and *K. oxytoca* producing KPC-33 and IMP-4 were misidentified as carbapenemase negative. Our findings indicate that the APB-EDTA method has a very low detection rate for isolates containing the IMP-4-type carbapenemase gene, with a sensitivity of only 9% (1/11) for isolates producing only IMP-4 and a sensitivity of 60% (3/5) for isolates producing both IMP-4 and KPC-2. Conversely, the REL/DPA/AVI method demonstrated excellent performance comparable to mCIM/eCIM in detecting isolates producing IMP-4, with 100% (16/16) sensitivity. Additionally, the REL/DPA/AVI method maintains comparable per-test costs (<$1) to both APB-EDTA method (<$1) and mCIM/eCIM (<$1), while demonstrating significant time efficiency by yielding results 16–18 h earlier than these two conventional methods. Operationally, unlike the two-step incubation process required by mCIM/eCIM, the REL/DPA/AVI method shares operational simplicity with the APB-EDTA method, requiring only a single incubation step following reagent addition. Furthermore, for carbapenemase-type identification, the REL/DPA/AVI method can be directly applied to positive blood cultures (requiring two-step differential centrifugation), bypassing the subculture and overnight incubation required by conventional phenotypic assays. However, to achieve a shorter liquid culture incubation time, the REL/DPA/AVI method necessitates the use of a shaking incubator.

**TABLE 4:** Comparison of the REL/DPA/AVI method with traditional phenotypic testing methods^*a*^

Test parameter	mCIM/eCIM	APB-EDTA method	REL/DPA/AVI method
Carbapenem-resistant organisms	Enterobacterales*, Pseudomonas aeruginosa*	Enterobacterales	Enterobacterales
Types of specimens	Bacterial colony	Bacterial colony	Bacterial colony, positive blood cultures
Operability	Involves a two-stage incubation process	Easy-to-perform, commercially available reagents	Imipenem solution and LB broth require fresh preparation; a shaking incubator is required
Outcome interpretation	Measurement of inhibition zone diameter	Measurement of inhibition zone diameter	Measurement of optical density (1 or 1.5 h) or naked-eye detection (1.5 h)
Turnaround time	18–20 h	18–20 h	1.5–2 h
Cost projection	<1.00$	<1.00$ (in-house); 2$ (commercial)	<1.00$; if the imipenem solution is not used within 3 d, the cost increases
Regulatory status	CLSI	Chinese Expert Consensus	-

^*a*^ "-", no result. Table constructed based on reference (15).

## DISCUSSION

With the increasing global prevalence of CRE, with their strong transmission capabilities and high infection fatality rates, particularly among patients with bloodstream infections, the rapid identification of these strains and confirmation of their resistance mechanisms have become particularly urgent (4–7, 28). This will facilitate the implementation of appropriate empirical treatment protocols and optimize antibiotic stewardship, thereby reducing associated mortality rates, healthcare costs, and the risk of CRE transmission (29).

In 2017, mCIM was first included in the CLSI M100 27th Edition Supplement and further clarified in the 28th Edition that the combined use of eCIM could accurately identify the presence of carbapenemases and differentiate between serine and metallo-β-lactamases (16, 18). The CLSI working group conducted a multicenter study that confirmed the average sensitivity and specificity of mCIM to be 97% and 99%, respectively (18). Subsequently, it was clarified that the sensitivity and specificity of eCIM were both >90% (16). Our findings are comparable to these data, with the sensitivity and specificity of mCIM being 97.81% and 100%, respectively, whereas those of eCIM were 97.56% and 100%, respectively. Some studies have reported that eCIM may miss the detection of IMP-type isolates (30, 31). In our study, all IMP-type strains were accurately identified, possibly due to the different sensitivities of the various subtypes to meropenem or differences in bacterial genera. We did not include isolates that co-produced class A and B carbapenemases in our calculations, as eCIM does not apply to situations where multiple carbapenemases are co-produced.

In our study, all these co-producers exhibited positive results for serine β-lactamases in mCIM/eCIM testing. Conversely, our method can rapidly identify these co-producers and achieve sensitivity and specificity in carbapenemase detection and classification of metallo-β-lactamases that are equal to or surpass those of mCIM/eCIM. This expedited method is based on the direct analysis of positive blood cultures. Bacterial enrichment is achieved through a two-step differential centrifugation process, which eliminates the need for subculturing. Our protocol features a brief incubation period (1–1.5 h) with shaking, making it distinct from the mCIM/eCIM. The mCIM/eCIM requires multiple sequential steps, including (i) a 4 h disk immersion in bacterial suspension, (ii) transfer of the impregnated disks to Mueller-Hinton agar plates pre-inoculated with *E. coli* ATCC 25922, and (iii) an overnight incubation period for zone-of-inhibition interpretation. However, our method cannot distinguish between isolates with imipenem resistance due to non-carbapenemase mechanisms and those co-producing multiple carbapenemases, as no single enzyme inhibitor can inhibit the growth of these bacteria. This limitation restricts the application of our method to *Pseudomonas aeruginosa* carbapenemase typing. Notably, KPC-33 was undetected using any of the testing methods. KPC-33 may arise from mutations in KPC-2 under the selective pressure of ceftazidime-avibactam, a mutation that typically results in resistance to ceftazidime-avibactam, but sensitivity to carbapenems, particularly imipenem (32, 33). Therefore, these variants may be undetectable by phenotypic testing based on the hydrolysis of carbapenems (19).

Leveraging the ability of EDTA to inhibit metallo-β-lactamases and the ability of APB to inhibit class A and C serine β-lactamases, they have been successively used to differentiate carbapenemase types (34–36). In 2010, Tsakris et al. (37) first combined these two inhibitors and proposed and optimized the APB-EDTA method algorithm, which has been recommended by the Chinese Expert Consensus as a phenotypic method for carbapenemase laboratory detection (17). Previous studies have demonstrated that the APB-EDTA method exhibits good discrimination performance for class A carbapenemases (sensitivity and specificity are both >95%), while for metallo-β-lactamases, its sensitivity ranges between 82.61% and 100% (36, 38–40). Our results are roughly consistent with these data, except for the aforementioned KPC-2 variant. Only 2.5% (1/40) of KPC-2-type isolates were not detected. However, the detection results for IMP-4-type isolates were less satisfactory. We observed that the sensitivity of the APB-EDTA method was only 36.84% (7/19) for isolates containing IMP-4 and as low as 9% (1/11) for isolates producing only IMP-4. Similar studies support this result. Zhang et al. and Franklin et al. believed that the difference in results was due to different susceptibility disks (39, 41).

However, our findings demonstrated that the detection of IMP genes remains poor, even with imipenem disks. Notably, our method exhibited excellent performance in this regard, with a sensitivity of 100% (19/19) for the IMP genes. When compared to the APB-EDTA method, the REL/DPA/AVI method demonstrates significant advantages in terms of operational efficiency and cost-effectiveness, while maintaining procedural simplicity. Although both methods require the preparation of standardized bacterial suspensions and the addition of antibiotics and inhibitors, our protocol’s unique feature lies in its ability to directly test positive blood cultures through two additional differential centrifugation steps. From a cost perspective, our method is more economical, with a per-test cost of less than $1 USD, compared to the commercial APB-EDTA method, which costs $2 USD per test. Furthermore, our method can detect OXA-48-like carbapenemases separately, although its sensitivity still needs improvement (71.4% at 1 h of incubation, which increases to 85.7% at 1.5 h). Our analysis indicated that *Enterobacter cloacae* isolate producing OXA-181 was inhibited in the tubes containing AVI and DPA after 1 h of incubation, leading to both tubes being judged as “–”. However, this inhibition was transient: the bacteria began to proliferate massively in the tube containing DPA after 1.5 h of incubation, and its optical density exceeded the cutoff value for a “+” judgment. Yamada et al. reported that a strain of *K. pneumoniae* producing an OXA-48-like type was inhibited by different concentrations of various metalloenzyme inhibitors (42). However, this phenomenon was not observed when the colonies were used as test specimens. We speculate that this may be attributed to the impact of centrifugation on bacteria or insufficient bacterial quantity owing to blood impurities (e.g., erythrocytes and debris elevating the turbidity of McFarland standards). Finally, we must point out that an isolate of *K. pneumoniae* producing OXA-232 was undetected, which could potentially be resolved by reducing the concentration of imipenem or extending the incubation time. However, the sample size was insufficient to support these results.

Various rapid diagnostic tests have been developed to enhance the sensitivity and specificity of CPE detection and reduce detection time. Biochemical reaction-based methods including Carba NP, commercialized RAPIDEC Carba NP, and NitroSpeed-Carba NP Test detect carbapenemase activity directly through chromogenic reactions (43–45). Immunochromatographic techniques (e.g., NG-Test Carba 5, Carba PBP) utilize lateral flow devices to visually identify specific carbapenemase antigens, completing detection within 15 min (21, 23). Recent advances have enabled direct detection in clinical specimens, particularly in blood cultures. The Verigene gram-negative blood culture (BC-GN) system can identify five major carbapenemase genes (KPC, NDM, VIM, IMP, and OXA-48-like) within 2 h of blood culture positivity, requiring less than 10 min of manual operation and achieving a 100% detection rate (46). Similarly, the updated FilmArray Blood Culture Identification (BCID2) can detect common resistance genes, including carbapenemase genes, within 1 h using only four to five drops of positive blood culture fluid (47). While these molecular technologies are becoming preferred laboratory tools due to their high automation, rapid turnaround time (1–2 h), and excellent accuracy, their widespread adoption faces economic constraints. The reagent costs per test for Verigene BC-GN and FilmArray BCID reach $60–80 and over $100, respectively, with total costs further escalating when combined with depreciation expenses of dedicated equipment (48, 49). These economic challenges have prompted research into more cost-effective detection methods. Shaidullina et al. used MALDI-TOF MS to detect CPE directly in positive blood cultures (50). However, it could not distinguish between specific enzyme types, limiting its guidance for clinical treatment. Meier and Hamprecht improved existing methods (Carba NP and CIM) through a lysis protocol to directly detect positive blood cultures, although this required additional reagents and purification steps (26). Volland developed the BL-DetecTool device to detect clinical specimens using LFIAs directly. However, its detection cost (estimated to be >200 CNY) remains relatively high compared to the mCIM/eCIM and APB-EDTA methods, particularly in low- and middle-income countries, posing a significant challenge (23, 39).

In this study, we developed a novel targeted carbapenemase phenotypic detection method that capitalizes on the differential growth status of CPE under the influence of enzyme inhibitors combined with antibiotics, using early optical density measurements for identification and judgment. Considering that imipenem has a faster bactericidal rate against bacteria such as *E. coli* and *Pseudomonas aeruginosa* than meropenem, this contributes to the rapid differentiation of bacterial growth states (51, 52). Although APB and EDTA have been widely used as β-lactam inhibitors in carbapenemase classification detection, our study revealed that low concentrations of APB combined with imipenem were insufficient to inhibit the growth of class A carbapenemase bacteria. Conversely, high concentrations of APB produced non-specific inhibitory effects on bacterial growth over a certain period, thereby prolonging the detection time. EDTA presented similar issues, even exhibiting non-specific bactericidal effects when combined with imipenem. Therefore, this study selected relebactam and DPA as alternatives to traditional inhibitors and included avibactam sodium to differentiate OXA-48-like carbapenemases (53–55). This method can rapidly produce results using liquid culture under suitable temperature and agitation conditions, significantly reducing the detection time compared with solid culture media and allowing the direct use of bacterial pellets from positive blood cultures after differential centrifugation as test samples.

Our study has several limitations. First, the validation process relied on contrived (seeded) blood culture specimens due to the low local prevalence of target carbapenemase genotypes, which may not fully represent real clinical scenarios. Future studies should prioritize validation using clinical specimens, and we encourage independent verification by peer laboratories. Second, the method’s dependence on shaking incubators presents equipment-specific constraints. While these devices are not universally available in routine laboratories, their cost remains significantly lower than molecular diagnostic platforms. When considering the clinical benefits of shortened detection time and improved antimicrobial stewardship, this methodology demonstrates favorable cost-effectiveness despite its equipment requirements.

In conclusion, the development of the REL/DPA/AVI method successfully balances analytical performance, operational simplicity, cost-effectiveness, and turnaround time. The method achieves sensitivity and specificity comparable to mCIM/eCIM while maintaining procedural complexity similar to the APB-EDTA method. Notably, its per-test cost (<$1 USD) is more economical than commercial APB-EDTA kits ($2 USD) while matching the turnaround time (1.5–2 h) of molecular diagnostics. The dual-interpretation capability of the method (visual observation or OD measurement) enhances its clinical utility for rapid CPE identification and enzyme type characterization, making it adaptable to diverse laboratory settings.

## SUPPLEMENTAL MATERIAL

Figure S1 jcm.01960-24-s0001.tif Optical density distribution at 1 and 1.5 h of incubation.

Supplemental figure legend jcm.01960-24-s0002.docx Figure S1 legend.

Supplemental tables jcm.01960-24-s0003.docx Tables S1 to S3.

ASM does not own the copyrights to Supplemental Material that may be linked to, or accessed through, an article. The authors have granted ASM a non-exclusive, world-wide license to publish the Supplemental Material files. Please contact the corresponding author directly for reuse.
