# Supplementary figures and images for: REL/DPA/AVI method: a novel approach for rapid detection of carbapenemase-producing Enterobacterales directly from positive blood cultures based on optical density
Source: J Clin Microbiol. 2025 May 12;63(6):e01960-24. doi: 10.1128/jcm.01960-24 (PMC12153349; doi:10.1128/jcm.01960-24)

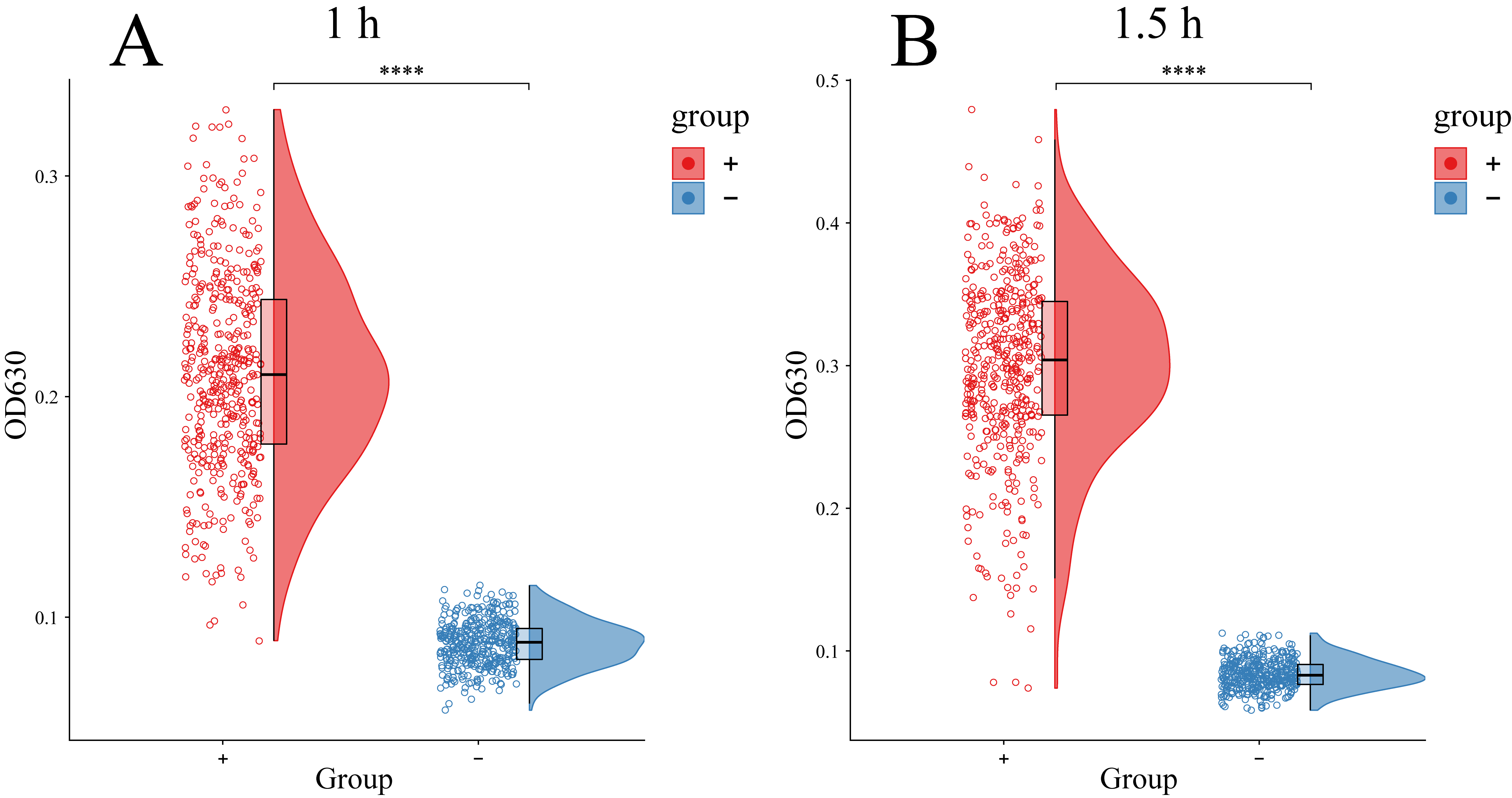

Supplement: Figure S1 — Optical density distribution at 1 and 1.5 h of incubation. [file jcm.01960-24-s0001.tif]
