# Supplementary material for: REL/DPA/AVI method: a novel approach for rapid detection of carbapenemase-producing Enterobacterales directly from positive blood cultures based on optical density
Source: J Clin Microbiol. 2025 May 12;63(6):e01960-24. doi: 10.1128/jcm.01960-24 (PMC12153349; doi:10.1128/jcm.01960-24)
Supplement: Supplemental figure legend — Figure S1 legend. [file jcm.01960-24-s0002.docx]

**Figure S1**. Optical density distribution at 1 and 1.5 h incubation. (A) Optical density (OD_630nm_) distribution of clinical isolates under growth (red) versus inhibition (blue) conditions at 1 h incubation. (B) Optical density (OD_630nm_) distribution of clinical isolates under growth (red) versus inhibition (blue) conditions at 1 h incubation. Statistical significance was determined by Mann-Whitney U test.

Note: “+”, growth; “-”, inhibition; h, hour; OD_630_, optical density at 630 nm; “****”, *P*-value is less than 0.001.
