# Supplementary material for: REL/DPA/AVI method: a novel approach for rapid detection of carbapenemase-producing Enterobacterales directly from positive blood cultures based on optical density
Source: J Clin Microbiol. 2025 May 12;63(6):e01960-24. doi: 10.1128/jcm.01960-24 (PMC12153349; doi:10.1128/jcm.01960-24)
Supplement: Supplemental tables — Tables S1 to S3. [file jcm.01960-24-s0003.docx]

**Table S1.** ROC parameters for optical density at 1 and 1.5 h incubation.

| Time (h) | AUC (95% CI) | Cutoff value | Youden Index | Sensitivity (%) | Specificity (%) | *P* value^a^ |
| --- | --- | --- | --- | --- | --- | --- |
| 1 | 0.998 (0.996–1.000) | 0.115 | 0.991 | 99.1 | 100 | 0.116 |
| 1.5 | 0.995 (0.989–1.000) | 0.114 | 0.993 | 99.3 | 100 |  |

Note: AUC, area under the curve; CI, confidence interval; a, using DeLong's test.

**Table S2.** Clinical isolates detected by the REL/DPA/AVI method and detection results.

| Carbapenemase type （no.） | Species | *β*-Lactamase(s) (no.) | Result | | | | | | | |
| --- | --- | --- | --- | --- | --- | --- | --- | --- | --- | --- |
|  |  |  | LB | | IPM and REL | | IPM and DPA | | IPM and AVI | |
|  |  |  | 1h | 1.5h | 1h | 1.5h | 1h | 1.5h | 1h | 1.5h |
| Carbapenemase producers (n = 137) |  |  |  |  |  |  |  |  |  |  |
| Class A (n=41) | *Klebsiella pnenmoniae* | KPC-2 (31) | + | + | - | - | + | + | - | - |
|  |  | KPC-33 (1) | + | + | - | - | - | - | - | - |
|  | *Klebsiella aerogenes* | KPC-2 (7) | + | + | - | - | + | + | - | - |
|  | *Enterobacter cloacae complex* | KPC-2 (1) | + | + | - | - | + | + | - | - |
|  | *Klebsiella pnenmoniae* | ATCC 1705 (1) | + | + | - | - | + | + | - | - |
| Class B (n=82) |  |  |  |  |  |  |  |  |  |  |
| NDM | *Klebsiella pnenmoniae* | NDM-1 (4) | + | + | + | + | - | - | + | + |
|  |  | NDM-3 (1) | + | + | + | + | - | - | + | + |
|  |  | NDM-5 (4) | + | + | + | + | - | - | + | + |
|  |  | NDM-16b (1) | + | + | + | + | - | - | + | + |
|  |  | NDM-51 (1) | + | + | + | + | - | - | + | + |
|  | *Escherichia coli* | NDM-1 (2) | + | + | + | + | - | - | + | + |
|  |  | NDM-3 (1) | + | + | + | + | - | - | + | + |
|  |  | NDM-4 (1) | + | + | + | + | - | - | + | + |
|  |  | NDM-5 (22) | + | + | + | + | - | - | + | + |
|  |  | NDM-15 (1) | + | + | + | + | - | - | + | + |
|  | *Klebsiella oxytoca* | NDM-1 (4) | + | + | + | + | - | - | + | + |
|  | *Klebsiella aerogenes* | NDM-1 (1) | + | + | + | + | - | - | + | + |
|  |  | NDM-5 (1) | + | + | + | + | - | - | + | + |
|  | *Enterobacter cloacae complex* | NDM-1 (15) | + | + | + | + | - | - | + | + |
|  |  | NDM-5 (5) | + | + | + | + | - | - | + | + |
|  | *Citrobacter freundii* | NDM-1 (2) | + | + | + | + | - | - | + | + |
|  | *Citrobacter braakii* | NDM-5 (1) | + | + | + | + | - | - | + | + |
|  | *Klebsiella pnenmoniae* | ATCC 2146 (1) | + | + | + | + | - | - | + | + |
| IMP | *Klebsiella pnenmoniae* | IMP-4 (9) | + | + | + | + | - | - | + | + |
|  | *Klebsiella oxytoca* | IMP-4 (2) | + | + | + | + | - | - | + | + |
| IMP+NDM | *Klebsiella pnenmoniae* | NDM-1+IMP-4 (3) | + | + | + | + | - | - | + | + |
| Class D (n=7) | *Klebsiella pnenmoniae* | OXA-48 (1) | + | + | + | + | + | + | - | - |
|  |  | OXA-232 (3) | + | + | + | + | + | + | - | - |
|  |  | OXA-232 (1) | + | + | - | - | - | - | - | - |
|  | *Enterobacter cloacae complex* | OXA-181 (1) | + | + | + | + | - | + | - | - |
|  | *Klebsiella pnenmoniae* | ATCC 2524 (1) | + | + | + | + | + | + | - | - |
| Double carbapenemases (n=7) | *Klebsiella pnenmoniae* | KPC-2+NDM-1 (2) | + | + | + | + | + | + | + | + |
|  |  | KPC-2+IMP-4 (4) | + | + | + | + | + | + | + | + |
|  | *Klebsiella oxytoca* | KPC-2+IMP-4 (1) | + | + | + | + | + | + | + | + |
| Non-carbapenemase  Producers (n=76) |  |  |  |  |  |  |  |  |  |  |
|  | *Klebsiella pnenmoniae* | CMY-170+TEM-179 (1) | + | + | - | - | - | - | - | - |
|  |  | CTX-M-73+OKP-A-11+CMY-170+TEM-207 (1) | + | + | - | - | - | - | - | - |
|  |  | CTX-M-73+SHV-33+CMY-170+TEM-207 (1) | + | + | - | - | - | - | - | - |
|  |  | CTX-M-73+SHV-75+TEM (1) | + | + | - | - | - | - | - | - |
|  |  | CTX-M-73+SHV-81+CMY-170+TEM-207 (1) | + | + | - | - | - | - | - | - |
|  |  | OKP-A-11+CMY-170+TEM-207 (3) | + | + | - | - | - | - | - | - |
|  |  | OKP-B-15+TEM-141 (1) | + | + | - | - | - | - | - | - |
|  |  | OKP-B-18+CMY-170+TEM-207 (1) | + | + | - | - | - | - | - | - |
|  |  | OXA-1+SHV-11 (2) | + | + | - | - | - | - | - | - |
|  |  | OXA-1+SHV-28+SHV-106+TEM-1+CTX-M-15 (1) | + | + | - | - | - | - | - | - |
|  |  | LEN-16 (1) | + | + | - | - | - | - | - | - |
|  |  | SHV-1+TEM-207 (1) | + | + | - | - | - | - | - | - |
|  |  | SHV-106+CMY-170+TEM-207 (1) | + | + | - | - | - | - | - | - |
|  |  | SHV-106+TEM-207 (1) | + | + | - | - | - | - | - | - |
|  |  | SHV-11+CMY-170+TEM-207 (1) | + | + | - | - | - | - | - | - |
|  |  | SHV-11+TEM-1 (1) | + | + | - | - | - | - | - | - |
|  |  | SHV-12+TEM-1+CTX-M-14 (1) | + | + | - | - | - | - | - | - |
|  |  | SHV-148+TEM-207 (2) | + | + | - | - | - | - | - | - |
|  |  | SHV-182+CMY-170+TEM-207 (1) | + | + | - | - | - | - | - | - |
|  |  | SHV-190+TEM-207 (2) | + | + | - | - | - | - | - | - |
|  |  | SHV-28+SHV-106+TEM-1+CTX-M-3 (1) | + | + | - | - | - | - | - | - |
|  |  | SHV-33+TEM-207 (1) | + | + | - | - | - | - | - | - |
|  |  | SHV-61+CMY-170+TEM-207 (1) | + | + | - | - | - | - | - | - |
|  |  | SHV-71+CMY-170 (1) | + | + | - | - | - | - | - | - |
|  |  | SHV-75+CMY-170+TEM-207 (1) | + | + | - | - | - | - | - | - |
|  |  | SHV-75+TEM-141 (1) | + | + | - | - | - | - | - | - |
|  |  | SHV-81+CMY-170+TEM-207 (1) | + | + | - | - | - | - | - | - |
|  |  | SHV-81+TEM-207 (1) | + | + | - | - | - | - | - | - |
|  |  | SHV-82+CMY-170+TEM-207 (1) | + | + | - | - | - | - | - | - |
|  |  | SHV-89+CMY-170+TEM-207 (2) | + | + | - | - | - | - | - | - |
|  |  | SHV-11+TEM-209+CTX-M-27+CTX-M-55 (1) | + | + | + | + | + | + | + | + |
|  | *Escherichia coli* | CMY-2+TEM-207 (1) | + | + | - | - | - | - | - | - |
|  |  | CTX-M-27 (1) | + | + | - | - | - | - | - | - |
|  |  | CTX-M-55 (1) | + | + | - | - | - | - | - | - |
|  |  | CTX-M-65+TEM-141 (2) | + | + | - | - | - | - | - | - |
|  |  | CTX-M-73+TEM-207 (1) | + | + | - | - | - | - | - | - |
|  |  | SHV-106+TEM-207 (1) | + | + | - | - | - | - | - | - |
|  |  | SHV-12+CMY-170 (1) | + | + | - | - | - | - | - | - |
|  |  | SHV-148 (1) | + | + | - | - | - | - | - | - |
|  |  | SHV-148+TEM-207 (1) | + | + | - | - | - | - | - | - |
|  |  | TEM-141 (2) | + | + | - | - | - | - | - | - |
|  |  | TEM-168 (1) | + | + | - | - | - | - | - | - |
|  |  | TEM-207 (12) | + | + | - | - | - | - | - | - |
|  |  | TEM-207+CMY-170 (3) | + | + | - | - | - | - | - | - |
|  | *Klebsiella oxytoca* | NR (1) | + | + | - | - | - | - | - | - |
|  | *Enterobacter cloacae complex* | TEM-207 (1) | + | + | - | - | - | - | - | - |
|  |  | NR (3) | + | + | - | - | - | - | - | - |
|  | *Citrobacter freundii* | CTX-M-73+CMY-170 (1) | + | + | - | - | - | - | - | - |
|  |  | TEM-207+CMY-115 (1) | + | + | - | - | - | - | - | - |
|  | *Pantoea agglomerans* | SHV-12 (1) | + | + | - | - | - | - | - | - |
|  |  | NR (1) | + | + | - | - | - | - | - | - |
|  | *Pantoea dispersa* | NR (1) | + | + | - | - | - | - | - | - |
|  | *Klebsiella pnenmoniae* | ATCC 1706 (1) | + | + | - | - | - | - | - | - |

Note: LB, LB broth; IPM, imipenem; REL, relebactam; DPA, dipicolinic acid; AVI, avibactam sodium; h, hour; NR, no result; “+”, growth; “-”, inhibition.

**Table S3.** Results of two phenotypic detection methods for CNSE.

| β-Lactamase(s) | No. | APB-EDTA method | | | | | mCIM/eCIM | | | |
| --- | --- | --- | --- | --- | --- | --- | --- | --- | --- | --- |
|  |  | A | B | A + B | Uncert | Neg | Ser | Met | Neg | Uncert |
| **KPC** |  |  |  |  |  |  |  |  |  |  |
| KPC-2 | 39 | 38 |  |  | 1 |  | 39 |  |  |  |
| KPC-33 | 1 |  |  |  |  | 1 |  |  | 1 |  |
| ATCC 1705 | 1 | 1 |  |  |  |  | 1 |  |  |  |
| **NDM** |  |  |  |  |  |  |  |  |  |  |
| NDM-1 | 28 |  | 28 |  |  |  | 1 | 26 | 1 |  |
| NDM-3 | 2 |  | 2 |  |  |  |  | 2 |  |  |
| NDM-4 | 1 |  | 1 |  |  |  |  | 1 |  |  |
| NDM-5 | 33 |  | 33 |  |  |  |  | 33 |  |  |
| NDM-15 | 1 |  | 1 |  |  |  |  | 1 |  |  |
| NDM-16b | 1 |  | 1 |  |  |  |  | 1 |  |  |
| NDM-51 | 1 |  | 1 |  |  |  |  | 1 |  |  |
| ATCC 2146 | 1 |  | 1 |  |  |  |  | 1 |  |  |
| **IMP** |  |  |  |  |  |  |  |  |  |  |
| IMP-4 | 11 |  | 1 |  |  | 10 |  | 11 |  |  |
| **NDM + IMP** |  |  |  |  |  |  |  |  |  |  |
| NDM-1 + IMP-4 | 3 |  | 3 |  |  |  |  | 3 |  |  |
| **OXA-48** |  |  |  |  |  |  |  |  |  |  |
| OXA-48 | 1 |  |  |  | 1 |  | 1 |  |  |  |
| OXA-181 | 2 |  | 1 |  | 1 |  | 1 |  | 1 |  |
| OXA-232 | 3 |  | 1 |  | 2 |  | 3 |  |  |  |
| ATCC 2524 | 1 |  |  |  | 1 |  | 1 |  |  |  |
| **Double carbapenemases** |  |  |  |  |  |  |  |  |  |  |
| KPC-2 + NDM-1 | 2 |  |  | 2 |  |  | 2 |  |  |  |
| KPC-2 + IMP-4 | 5 |  |  | 3 | 2 |  | 5 |  |  |  |
| **Non-carbapenemase**  **Producers** |  |  |  |  |  |  |  |  |  |  |
| OXA-1 + SHV-11 | 1 |  |  |  |  | 1 |  |  | 1 |  |
| SHV-11 + TEM-209 + CTX-M-27 + CTX-M-55 | 1 |  |  |  |  | 1 |  |  | 1 |  |
| CTX-M-55 | 1 |  |  |  |  | 1 |  |  | 1 |  |
| ATCC 1706 | 1 |  |  |  |  | 1 |  |  | 1 |  |
| **Total** | 141 | 39 | 74 | 5 | 8 | 15 | 54 | 80 | 7 | 0 |

Note: A, Class A carbapenemases;B, Class B carbapenemases; D, Class D carbapenemases; A + B, co-producer of Class A and B carbapenemases; Neg, carbapenemase-negative; Ser, serine-β-lactamase; Met, metallo-β-lactamase; ATCC 1705, *K. pnenmoniae* ATCC BAA-1705; ATCC 1706, *K. pnenmoniae* ATCC BAA-1706; ATCC 2146, *K. pnenmoniae* ATCC BAA-2146; ATCC 2524, *K. pnenmoniae* ATCC BAA-2524.
